# Chromosome painting in the manatee supports Afrotheria and Paenungulata

**DOI:** 10.1186/1471-2148-7-6

**Published:** 2007-01-23

**Authors:** Margaret E Kellogg, Sandra Burkett, Thomas R Dennis, Gary Stone, Brian A Gray, Peter M McGuire, Roberto T Zori, Roscoe Stanyon

**Affiliations:** 1 College of Veterinary Medicine, University of Florida, PO BOX 100245, Gainesville, FL 32610-0245, USA; 2 Comparative Molecular Cytogenetics Core, National Cancer Institute, Frederick, MD 21702, USA; 3 Department of Pediatrics, Division of Genetics, University of Florida, PO Box 100296, UFHSC, Gainesville, FL 32610, USA; 4 Department of Biochemistry and Molecular Biology, University of Florida, PO Box 100245, College of Medicine, Gainesville, FL 32610, USA; 5 Department of Animal Biology and Genetics, University of Florence, Florence, Italy, Via del Proconsolo 12, 50122 Florence, Italy (formerly at NCI, Frederick)

## Abstract

**Background:**

Sirenia (manatees, dugongs and Stellar's sea cow) have no evolutionary relationship with other marine mammals, despite similarities in adaptations and body shape. Recent phylogenomic results place Sirenia in Afrotheria and with elephants and rock hyraxes in Paenungulata. Sirenia and Hyracoidea are the two afrotherian orders as yet unstudied by comparative molecular cytogenetics. Here we report on the chromosome painting of the Florida manatee.

**Results:**

The human autosomal and X chromosome paints delimited a total of 44 homologous segments in the manatee genome. The synteny of nine of the 22 human autosomal chromosomes (4, 5, 6, 9, 11, 14, 17, 18 and 20) and the X chromosome were found intact in the manatee. The syntenies of other human chromosomes were disrupted in the manatee genome into two to five segments. The hybridization pattern revealed that 20 (15 unique) associations of human chromosome segments are found in the manatee genome: 1/15, 1/19, 2/3 (twice), 3/7 (twice), 3/13, 3/21, 5/21, 7/16, 8/22, 10/12 (twice), 11/20, 12/22 (three times), 14/15, 16/19 and 18/19.

**Conclusion:**

There are five derived chromosome traits that strongly link elephants with manatees in Tethytheria and give implicit support to Paenungulata: the associations 2/3, 3/13, 8/22, 18/19 and the loss of the ancestral eutherian 4/8 association. It would be useful to test these conclusions with chromosome painting in hyraxes. The manatee chromosome painting data confirm that the associations 1/19 and 5/21 phylogenetically link afrotherian species and show that Afrotheria is a natural clade. The association 10/12/22 is also ubiquitous in Afrotheria (clade I), present in Laurasiatheria (clade IV), only partially present in Xenarthra (10/12, clade II) and absent in Euarchontoglires (clade III). If Afrotheria is basal to eutherians, this association could be part of the ancestral eutherian karyotype. If afrotherians are not at the root of the eutherian tree, then the 10/12/22 association could be one of a suite of derived associations linking afrotherian taxa.

## Background

Recently the molecular based approaches of super-ordinal grouping of extant eutherians (Afrotheria, Euarchontoglires, Laurasiatheria and Xenarthra) has gained popularity [1-3]. However, one of the four proposed super-orders, Afrotheria, is controversial because it unites morphologically distinct species of African placentals (golden moles, tenrecs, otter shrews, elephant shrews, aardvarks, hyraxes, elephants and sirenians). Within Afrotheria, sirenians, elephants and hyraxes form a clade called Paenungulata. There is little morphological or paleontological evidence that provides support for Afrotheria [4]. A movable snout was hypothesized as a synapomorphic trait, but this feature is apparently not homologous across different afrotherian lineages[5]. More recently, it was proposed that aspects of placentation could provide a synapomorphy for this assemblage [6,7]. Some outstanding issues in higher eutherian phylogenomics include the exact root of the placental tree, the relationships within the super-ordinal clade Laurasiatheria (moles, hedgehogs, shrews, bats, cetaceans, ungulates, pangolins and carnivores), and resolving the trichotomy of sirenians, elephants and hyraxes [8].

Sirenia and Hyracoidea are the two afrotherian orders remaining to be investigated with molecular cytogenetic techniques. In this paper, the chromosome painting of the Florida manatee (*Trichechus manatus latirostris*) is reported. These data should be a valuable addition to our understanding of afrotherian relationships and the eutherian ancestral karyotype.

### The Florida manatee

The endangered Florida manatee is a subspecies of the West Indian manatee (*Trichechus manatus*) in the order Sirenia. Sirenians are often considered phylogenetic outliers. Despite similarities in adaptations, habitat, and body shape, they have no evolutionary relationship with the other orders of marine mammals. Extant sirenians are the only herbivorous marine mammals and live in fresh, brackish or marine habitats dispersed along tropical and subtropical environments.

### Previous cytogenetic reports on manatees

Solid stained chromosome studies were completed on a limited number of individual manatees, establishing the chromosome number as 2N = 48 for the Florida manatee [9,10] and 2N = 56 for the Amazonian manatee (*Trichechus inunguis*) [11]. Following solid staining, chromosome-banding procedures allowed for the identification of individual chromosome regions. Giemsa and trypsin staining, or GTG-banding, was used to create karyotypes and ideograms for the Florida manatee [12] and the Amazonian manatee [13].

Comparisons of chromosome painting data provide an independent test of the contrasting hypotheses on mammalian evolution and phylogeny. The research presented here clarifies the phylogenetic position of the manatee and tests the validity of the radical taxonomic assemblage known as Afrotheria. The results are then compared to other chromosome painting data in Afrotheria. In light of the findings, the relationships within Afrotheria and the alternative organizations of the ancestral eutherian karyotype are assessed.

## Results

**Figure 1:**
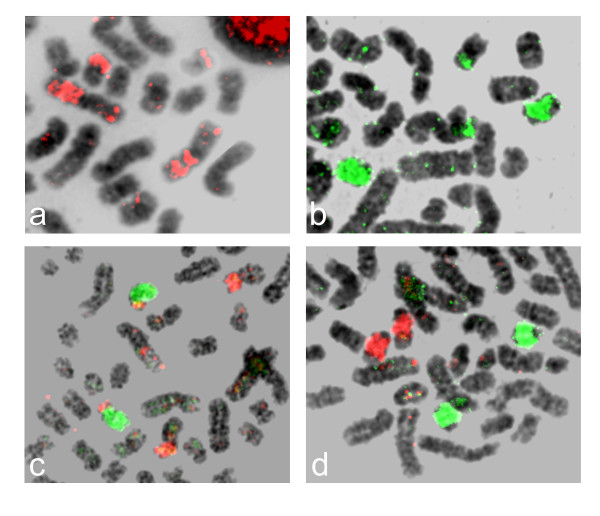
Examples of hybridizations in the manatee a) human 12, b) human 13, c) human 14 in green and 15 in red d) human 17 in green and 18 in red.

**Figure 2:**
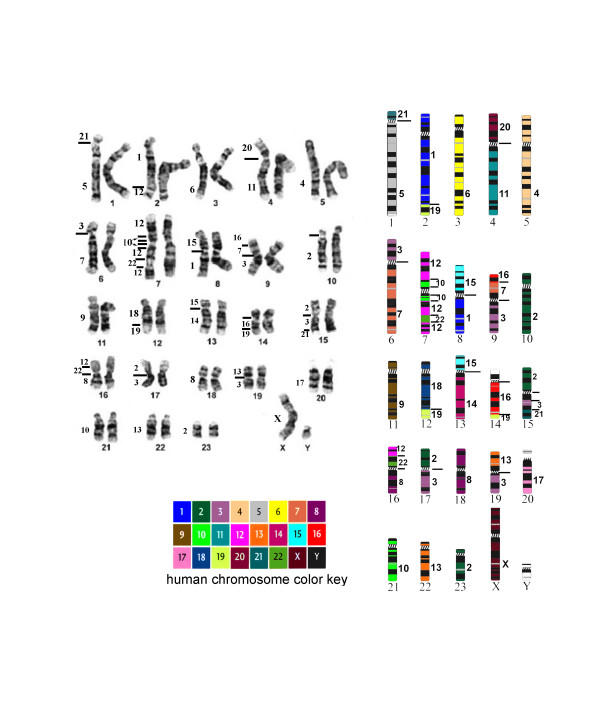
The karyotype of the manatee is shown to the left and the color coded idiogram to the right (modified from Gray et al. 2002). Manatee chromosomes are numbered below and human chromosome homology is shown laterally.

Examples of human chromosome paints (HSA) hybridized to manatee (TMA) metaphase chromosomes are shown in Figure 1. Synteny was found intact in nine (4, 5, 6, 9, 11, 14, 17, 18 and 20) of the 22 human autosomal and X chromosomes (Figure 2). Two hybridization signals were evident on separate manatee chromosomes for ten human chromosomes (1, 7, 8, 10, 12, 13, 15, 16, 21 and 22). The human 19 paint hybridized to three TMA chromosomes (2, 12 and 14). Human chromosomes 2 and 3 were highly fragmented in the manatee genome and painted four and five chromosomes, respectively (Table 1). Due to the small signals involved and the quality of the metaphases, it was more difficult to assign the hybridization pattern for these two chromosomes. Human chromosome paint 12 provided three signals on TMA 7, most likely due to an inversion. Chromosome paints with pericentromeric signals on both arms of the same chromosome were considered as one signal. Centromere areas on the manatee karyotype were not hybridized. The Y chromosome was the only human probe that failed to provide a signal in the manatee. Altogether, the human autosomal chromosome paints and the X chromosome paint delimited a total of 44 homologous segments in the manatee genome. Human chromosome paints hybridized to 20 (15 unique) segments in the manatee genome: 1/15, 1/19, 2/3 (twice), 3/7 (thrice), 3/13, 3/21, 5/21, 7/16, 8/22, 10/12 (twice), 11/20, 12/22 (thrice), 14/15, 16/19 and 18/19.

**Table 1:** 

	2n	1	2	3	4	5	6	7	8	9	10	11	12	13	14	15	16	17	18	19	20	21	22
AEK	48	1	2	1	1	1	1	2	2	1	2	1	2	1	1	1	2	1	1	2	1	1	2
manatee	48	2	4	5	1	1	1	2	2	1	2	1	2(4)	2	1	2	2	1	1	3	1	2	2
golden mole	30	1	2	1	1	1	1	2	2	1	2	1	2	1	1	1	2	1	1	2	1	1	2
elephant shrew	26	1	2	4	1	1	1	2	2	1	2	1	2	1	1	1	2(3)	1	1	2(3)	1	1	2
aardvark	20	1	2	1	1	1	1	2	2	1	2	1	2	1	1	1	2	1	1	2	1	1	2
elephant	56	4	4	5	3	1	1	2	2	1	2	3	2	2	1	2	2	1	1	3	1	2	2

Number of segments homologous to human chromosome found in Afrotheria species. The taxa in the first, left column: AEK = ancestral eutherian karyotype [14, 16, 40]. The second column list the 2n, diploid numbers for each species and the remaining columns refer to signals found for each human chromosome. The number in brackets refers to higher number of hybridization signals due to pericentric inversions.

## Discussion

The painting map of the manatee genome was compared with results published on other Afrotheria taxa: aardvark, elephant, elephant shrew and golden mole [14-17]. An assessment of the associations found in each taxa are shown in Table 1. All species have eight associations in common (1/19, 3/21, 5/21, 7/16, 10/12, 12/22, 14/15 and 16/19). Five of these associations are considered ancestral to all eutherians by most proposals (3/21, 7/16, 12/22 twice, 14/15 and 16/19). It appears that the associations 1/19 and 5/21 can be used to link afrotherian species [14-16,18]. These associations provide cytogenetic support, in agreement with molecular studies, that Afrotheria is a natural clade.

New chromosome painting data in Xenarthra (anteaters, sloths and armadillos) are also informative towards the ancestral eutherian karyotype. Of the four species studied, *Tamandua tetradactyla, Choloepus didactylus, C. hoffmanii *and *Dasypus novemcinctus *[18,19], only the anteater has a 1/19 association. It is not likely that this association is homologous to Afrotheria, because the anteater has the most highly rearranged karyotype known in Xenarthra [18].

The manatee data indicate that the association 10/12/22 is most likely ubiquitous throughout Afrotheria. A combination HSA10p/12p/22q and a single HSA10q were found in the aardvark and elephant karyotypes [14,17]. An apparently identical association was later found in the elephant shrew and golden mole [15]. The question is, whether this association is a third cytogenetic landmark for the Afrotheria clade, or instead should be considered part of the ancestral eutherian karyotype.

The entire 10/12/22 association appears to be present in clades I, Afrotheria, and IV, Laurasiatheria, only partially present in clade II, Xenarthra (10/12), and absent in clade III, Euarchontoglires (primates, rabbits, rodents, tree shrews and flying lemurs). Carnivores have a homologous 10/12/22 association to Afrotheria, as demonstrated by reciprocal chromosome painting [20,21]. Eulipotyphla (shrews, solenodons, moles, hedgehogs, and *Nesophontes*) also have the 10/12/22 association [19,22]. Chromosome painting data in Xenarthra show that a 10/12 association is present in the armadillo (*D. novemcinctus*) [18]. To date, the 10/12 association has been found in three of the four eutherian mammal clades. Yet, there is no reciprocal painting in Xenarthra to prove that the 10/12 association is truly homologous to that found in Afrotheria. Several hypotheses can be developed with different implications if Afrotheria or Xenarthra is considered basal. If Afrotheria is basal, the occurrence of 10/12/22 in clades I and IV would suggest that this association is part of the ancestral eutherian karyotype with a subsequent, independent loss in clades II and III. The occurrence of the 10/12/22 association clades I and IV, could be considered a phylogenetic link. Alternatively, the association could have been independently acquired in the two clades. If Xenarthra is basal, this association could have originated in Afrotheria and was then lost in clade III.

Association 3/13 was found in the manatee, elephant and elephant shrew. However, there are no reciprocal painting data between human and manatee or human and elephant shrew. Therefore, it is not possible to confirm that the 3/13 association is homologous (involves the same segments of both chromosomes 3 and 13). In view of the afrotherian molecular data, this association was independently derived in the Macroscelidae (elephant shrews) and Paenungulata phylogenetic lineages [8].

### Support for the Tethytheria and Paenungulata assemblage

Before the advent of molecular studies, some morphologists placed sirenians, elephants and hyraxes under Ungulata. Elephants and sirenians were grouped together in Tethytheria, while hyraxes were placed in Phenacodonta along with perissodactyls [23]. Results in molecular studies are inconsistent and fail to resolve the Paenungulate trifurcation [8] and some data do not support Tethytheria [2,24-26]. Mitochondrial genome analyses do support Tethytheria, but exclude Hyracoidea [1]. SINE insertion data produced incongruent phylogenetic relationships within Paenungulata, most likely due to a rapid divergence from a highly polymorphic last common ancestor [27].

The chromosome mapping data strongly support Tethytheria (sirenians and elephants) and implies support for the clade Paenungulata (Sirenia, Proboscidea, and Hyracoidea). There appear to be four derived associations linking elephants with manatees: 2/3, 3/13, 8/22 and 18/19. HSA 4/8p was not present in the manatee and may represent a derived trait of Paenungulata. Both publications on the elephant indicate that this association is also lacking [14,17]. It is possible that the 4/8 association went undetected in our study, as well as in elephants. Although, the widespread occurrence of the 4/8 association in all mammals, outside of elephants and most primates, lends credence to its inclusion in the ancestral eutherian karyotype. It would be useful to test these hypotheses with rock hyrax chromosome painting data.

### Branching order in Afrotheria

The branching order within Afrotheria has not reached a consensus. Some authors have viewed Macroscelidae, the elephant shrews, as the most basal and early divergent order within Afrotheria [2,28]. However, Murphy et al. (2001) placed the triumvirate of sirenians, elephants and hyraxes (Paenungulata) as basal, verified by additional molecular data [1,3,29]. It is difficult to determine which order is most basal because sirenians and elephants, like other afrotherian species, have fairly derived karyotypes.

According to Robinson et al. (2003), associations 2/8, 3/20 and 10/17 link elephant shrews, golden moles/tenrecs and aardvarks. Only the association 2/8 is present in all three. Recently, the association 2/8 was also found in anteater (*T. tetradactyla*), sloth (*Choloepus didactylus*) and pangolin (*Manis javanica*) [18,19]. Associations 3/20 and 10/17 are lacking in golden moles/tenrecs. Murphy et al. (2004) proposed that the associations 3/20 and 10/17 were probably lost in golden moles/tenrecs. No reciprocal painting was done in elephant shrews or golden moles/tenrecs and it is therefore unknown if these associations are actually homologous. There is weak cytogenetic evidence linking elephant shrews and golden moles/tenrecs. An alternate hypothesis might be a sister relationship between elephant shrews and aardvarks. Perhaps a rapid divergence in elephant shrews, golden moles/tenrecs and aardvarks also resulted in limited phylogenetic signals for these chromosome associations.

### The root of the Eutherian tree

Although the super-order assemblies appear well established, the most basal position on the eutherian tree has not been determined with certainty [2,25,30]. Afrotheria and Xenarthra are the two oldest eutherian clades and probably emerged from the Southern Hemisphere in excess of 100 million years ago [31,32]. Molecular dating and biogeography have provided evidence that crown-group Eutheria may have their most recent common ancestry in the Southern Hemisphere (Gondwana) [32]. The other two clades (Laurasiatheria and Euarchontoglires) can be grouped as Boreoeutheria [33].

There are currently three hypotheses for the root of the eutherian tree. Most discussions from molecular studies place emphasis on either Afrotheria or Xenarthra as the most basal clade [25,34]. A third hypothesis states that the ancestral eutherian karyotype is a combination of both clades. This hypothesis cannot be completely ruled out and is preferred in some studies [35,36]. However, the suite of derived chromosomal associations found in all studied Afrotheria argues against the hypothesis that a combination of the two clades is basal to the eutherians.

Recently, a report on retroelements gives support for the hypothesis that Xenarthra is the sister group to all other placentals [37]. Indeed, new cytogenetic comparisons show that the proposed ancestral eutherian karyotype is essentially conserved in Xenarthra, specifically in the two-toed sloth (*Choloepus hoffmanii*) [16]. These two studies should be given attention because both take into consideration rare genomic events in which convergence is particularly limited. The conserved xenarthran karyotype may well be indicative of their phylogenomic position among eutherians. However, an essential point is that all reconstructions of the ancestral eutherian karyotype are preliminary until a relevant outgroup is studied with chromosome painting. A taxonomically rich array of species supported by appropriate out-groups is vital to the reconstruction of mammalian genome evolution. The deficiency of comparative chromosome painting data between eutherians and marsupials is a severe limitation on attempts to delineate the mammalian ancestral genome. The analyses of other afrotherians, xenarthrans and marsupials may clarify these unresolved questions.

## Conclusion

The chromosome painting data presented here leave little doubt that Tethytheria is a clade within Afrotheria and implies support for the Paenungulata assemblage. Recent retroposon data also confirmed Paenungulata, but could not resolve the phylogenetic relationships among elephants, sirenians and hyraxes [27]. It is generally appreciated that characters with high evolutionary rates provide good phylogenetic resolution. Afrotherian karyotypes demonstrate high rates of chromosome evolution and numerous derived inter-chromosomal rearrangements link elephants and manatees. It is therefore likely that additional chromosome painting in rock hyraxes could shed light on the divergence sequence and resolve the Paenungulata trichotomy.

## Methods

Chromosome preparations of a male Florida manatee (*Trichechus manatus latirostris*, TMA) were obtained from peripheral blood mononuclear cells (PBMCs) and primary fibroblast cartilage cell culture. Cells were cultured in RPMI 1640 (Hyclone) supplemented with 20% fetal bovine serum (FBS), L-glutamine (0.01%) and gentamicin (25 μg/ml). PBMCs were incubated in-vivo using phytohemagglutinin (PHA, 0.25 mg/mL) as a mitotic stimulant for 72 to 96 hr at 36°C in 5% carbon dioxide, 95% air and 100% relative humidity. Routine procedures were used for chromosome preparations. We followed the chromosome nomenclature as previously published [12] pairing and grouping chromosomes by banding patterns, relative lengths and morphology.

Human chromosome paints were obtained as previously described by chromosome flow sorting followed by degenerate oligonucleotide primed PCR amplification [38,39]. Paints were labeled with either biotin-dUTP, digoxigen-dUTP (both from Roche Applied Science) or Spectrum Orange-dUTP (Vysis).

Interspecific *in-situ *hybridizations of Florida manatee chromosomes with human probes were performed with 300 to 500 ng of each biotin-labeled probe, 10 μg of human Cot-1 DNA and 5 μg of ssDNA. The mixture was precipitated and dissolved in 13–15 μl of hybridization mixture (formamide 50%, dextran sulfate 10%, 2 × SSC). Direct labeling with Spectrum Orange followed a Nick Translation protocol (Vysis) using 1 μg of each amplified human DNA probe, 0.2 mM Spectrum Orange and 25 μg each of human and manatee Cot-1 DNA (Applied Genetics Laboratories, Inc.). The mixture was precipitated and dissolved in 10 μl distilled water. Approximately 300 ng of probe from this mixture were dissolved in 10.5 μl Hybrizol VII (Q-BIOgene) and 0.75 μg each of human and manatee Cot-1 DNA.

The labeled probe mixture was denatured at 80°C for 10 min and reannealed at 37°C for 90 min before hybridization. Slides were aged at 37°C for 30 min followed by dehydration in a room temperature 70, 80, 90, and 100% ethanol series. The DNA was denatured in 70% formamide/2 × SSC, at 65°C for 90–120 s, and quenched in an ice-cold ethanol series. Hybridization was carried out in a humidity chamber at 37°C for five days. Post-hybridization washes followed standard procedures at 40°C. Biotin detection was performed with avidin-conjugated FITC (Vector) for 45 min at 37°C. Counterstaining was performed with DAPI (0.8 ng/μl) for 10 min and the slides were mounted with antifade (100 mg p-phenylenediamine in 80 ml glycerine, 20 ml PBS, pH 8).

Analyses were performed under a Zeiss Axiophot 2 or Axioskop fluorescence microscope coupled with a CCD camera (Photometrics), and images were captured with the Smart Capture software (Digital Scientific Inc.).

## Authors' contributions

RS and RZ conceived and designed the experiments. MK, SB, TD and RS performed the experiments. BG, MK, RZ, GS and PM prepared and contributed reagents/cell cultures/analysis tools. RS, MK and SB analysed the data. RS, MK and PM wrote the paper. All author read and approved the final manuscript.

**Table 2:** 

	1/19	2/3	2/8	3/13	3/20	3/21	4/8	5/21	7/16	8/22	10/12	10/17	12/22	14/15	16/19	18/19	total
AEK						X	X		X		?		X	X	X		6–7
elephant shrew	X		X	X	X	X	X	X	X		X	X	X	X	X		21
golden mole	X		X			X	X	X	X		X		X	X	X		14
Aardvark	X		X		X	X	X	X	X		X	X	X	X	X		20
Elephant	X	X		X		X		X	X	X	X		X	X	X	X	17
Manatee	X	X		X		X		X	X	X	X		X	X	X	X	15

Chromosomal associations found in two or more Afrotheria taxa. AEK = ancestral eutherian karyotype [14, 16, 40]. Associations were counted only once to derive the total.
